# Volunteering with mild cognitive impairment: implications for subsequent cognitive changes

**DOI:** 10.1093/geront/gnaf190

**Published:** 2025-09-10

**Authors:** Meng Huo, Kyungmin Kim

**Affiliations:** Department of Human Ecology, University of California, Davis, Davis, California, United States; Department of Child Development and Family Studies, College of Human Ecology, Seoul National University, Seoul, South Korea; Integrated Major in Regional Studies and Spatial Analytics, Seoul National University, Seoul, South Korea

**Keywords:** Health and Retirement Study, Cognitive impairment no Dementia, Volunteer, ADRD, Dementia

## Abstract

**Background and Objectives:**

Volunteering has cognitive benefits in later life and has been theorized to protect against Alzheimer’s disease and related dementias (ADRD). A small but growing body of volunteer programs target people with mild cognitive impairment (MCI)—who are presumably at elevated risk for ADRD, but we know surprisingly little about who volunteers with MCI and how volunteering affects their subsequent cognitive changes. The current study sought to address these gaps.

**Research Design and Methods:**

We used longitudinal data from the *Health and Retirement Study* (2002–2018) and identified a pooled sample of 6,930 midlife and older adults (aged 50+) who met criteria for MCI based on their cognitive scores on the Telephone Interview for Cognitive Status. We tracked these participants’ sociodemographic characteristics, volunteer activities, and cognitive scores in the subsequent 4 years.

**Results:**

A two-level logistic regression showed that among midlife and older adults with MCI, those who attained more years of education, had greater wealth, reported a history of volunteering, and had better self-rated health as well as fewer functional limitations were more likely to report volunteering in the presence of MCI. Volunteers with MCI—particularly those who continuously volunteered or initiated volunteering—exhibited more positive cognitive changes over time.

**Discussion and Implications:**

This study highlights the importance of socioeconomic resources and health in predicting volunteering with MCI and reveals lasting cognitive benefits of volunteering as midlife and older adults adjust to their cognitive impairment. Findings call for more tailored volunteer opportunities for people with MCI.

A large and increasing number of midlife and older adults are living with mild cognitive impairment (MCI)—noticeable memory and thinking problems that are not typical for their ages (Song et al., 2023; Ward et al., 2012). MCI has been commonly regarded as a precursor of Alzheimer’s disease and related dementias (ADRD). Nevertheless, the conversion does not necessarily occur (Bruscoli & Lovestone, 2004; Hu et al., 2017), which makes MCI a critical time window to slow cognitive declines and protect against ADRD. Scholars have proposed a variety of treatments to ameliorate symptoms in people with MCI, and nonpharmacological interventions are often highly regarded due to their minimal adverse side effects (Lissek & Suchan, 2021). Cognitive training programs may seem most intuitive, but their effects are typically domain-specific and short-term (Li et al., 2011; Reijnders et al., 2013). Instead, lifestyle interventions tend to promote social, physical, and cognitive activities on a more sustained basis and have proved to be more promising in benefiting people with MCI (Kivipelto et al., 2018).

To inform lifestyle interventions targeting people with MCI, the current study focused on volunteering—a prosocial behavior that has been consistently associated with cognitive benefits (Guiney & Machado, 2018; Kail & Carr, 2020; Sharifi et al., 2024). Volunteering typically takes place under the auspices of formal organizations and involves efforts to help people outside of volunteers’ social networks (Carr et al., 2015). MCI may present cognitive and functional obstacles that hinder older adults from engaging in volunteer activities that have been primarily designed for those who are physically able. Yet, volunteering can play central roles in fulfilling older adults’ needs for purpose in life and sustaining their sense of dignity in the context of MCI. A small but growing number of programs have been designed to target people with MCI and provide customized volunteer opportunities matched to their capabilities (e.g., teach English one-on-one via videoconferencing, run small errands within assisted living facilities; Klinedinst & Resnick, 2016; Piette et al., 2023a, 2023b). Empirical evidence, however, is scant, and studies have been primarily conducted with small, educated samples and focused on testing program feasibility. We know little about the social profile of older adults who volunteer in the context of MCI and the lasting cognitive benefits of their volunteer activities.

To address these gaps in the literature, we drew on a sample of midlife and older adults living with MCI from a nationally representative panel study, the *Health and Retirement Study* (HRS). The purpose of this study was to identify among people with MCI those who volunteered and track their longitudinal cognitive consequences. Findings can reveal risk and resilience as people adapt to MCI and shed light on the development of targeted and tailored volunteer programs that can protect against severe cognitive declines.

## Theoretical framework

To guide our study, we integrated the volunteer process model (Musick & Wilson, 2007; Omoto & Snyder, 2002; Wilson, 2012) with several additional theoretical perspectives. The volunteer process model offers a sociological perspective to delineate the antecedents, experience, and consequences of volunteering. When identifying the antecedents of volunteering, this model primarily considers subjective dispositions such as personality traits that *motivate* people to volunteer as well as resources that *enable* people to volunteer. As people engage in volunteer activities, their experience is not limited to just assuming the role of a volunteer but rather involves a dynamic process whereby volunteers transition in and out their roles. These experiences—routine, continuous engagement in volunteer activities—in turn lead to a variety of positive health outcomes, including cognitive benefits.

We expanded this model to account for the presence of MCI and drew on the ecological model of aging to reveal the social profile of volunteers with cognitive impairment (Lawton & Nahemow, 1973). The ecological model of aging highlights the interplay between personal competence (functional capacities) and environment press (demands and opportunities). As functional capacities decline in MCI, resources that can directly affect volunteer opportunities may have greater influences on people’s behaviors than subjective dispositions. Here, we primarily examined resources (e.g., wealth, health) to characterize midlife and older adults who volunteered while living with MCI.

Volunteering in this context provides midlife and older adults with a unique opportunity to stay active, connected, and cognitively stimulated (Renn et al., 2021), which is central as having MCI can compromise one’s identity as a useful and valued person (Carter et al., 2023; Williams et al., 2014). Activity theory has long proposed the psychosocial benefits of engaging in meaningful activities (Jarosz, 2022; Lemon et al., 1972), which was further theorized as mechanisms underlying the association between volunteering and reduced dementia risk among older adults (Anderson et al., 2014). Incorporating these perspectives, we proposed that volunteering would slow cognitive declines in midlife and older adults living with MCI.

## Resources associated with volunteering in MCI

We considered multiple indicators to capture resources that might affect volunteering in the context of MCI: socioeconomic status (SES), volunteering history, and physical and mental health. SES, as reflected by education, income, and wealth, has been consistently associated with volunteering (Choi & Chou, 2010; Dury et al., 2015; Tang, 2008). People with higher SES tend to have greater resources to identify and access volunteer opportunities, which may be particularly important as they manage MCI and need tailored programs matched to their reduced capabilities (Zhu et al., 2023). Indeed, extant research on volunteer programs designed for people with MCI have all focused on older adults with at least some college education (Klinedinst & Resnick, 2016; Piette et al., 2023b). As such, we expected midlife and older adults with upper SES would be more likely to volunteer in the presence of MCI.

Likewise, a prior history of volunteering may grant people access to a greater variety of programs, including those matched to their (compromised) capabilities. Volunteers with prior and extensive experience are less likely to stop volunteering (Tang et al., 2010). In fact, even participation in high school extracurricular activities predicts volunteering in mid to later life (Greenfield & Moorman, 2018). Engagement in volunteering is largely stable among midlife and older adults (Choi & Chou, 2010), which may not change even when they are adjusting to MCI and still able to live and function independently. Thus, among those with MCI, we expected prior volunteer experience to be associated with a greater likelihood of volunteering.

Physical and mental health are significant correlates of volunteering, presumably because volunteer activities can be somewhat physically or mentally demanding. Many studies propose and support causal paths whereby volunteering improves physical and mental health; yet, research does also suggest that poor physical health (e.g., worse self-rated health, more functional limitations, more chronic conditions) and mental health (e.g., more depressive symptoms) can be barriers to volunteering (Li & Ferraro, 2005, 2006). Among people with MCI, we expected physical and mental health to differentiate their engagement in volunteering.

## Volunteering and subsequent cognitive changes in MCI

We also examined how volunteering was associated with subsequent cognitive changes in people with MCI. Three reviews were conducted in the last decade to understand the health consequences of volunteering (Anderson et al., 2014; Guiney & Machado, 2018; Sharifi et al., 2024). Although the literature focused on volunteering and cognitive health is scant, all reviews concluded that midlife and older volunteers had better cognitive performance and were less likely to meet criteria for cognitive impairment than their nonvolunteer counterparts. Nevertheless, only one study to date examined volunteering and cognitive changes in people with MCI, who are presumably at elevated risk for developing dementia (Pandya et al., 2016). Hughes et al. (2013) tracked a sample of 816 older adults with MCI for 3 years and showed a greater percentage of volunteers (39.4%) in those who did not progress to severe cognitive impairment (*n *= 738) versus volunteers (24.4%) in those who did (*n *= 78). The comparison result should be interpreted with caution, though, in that the association between volunteering and progression from MCI was not a key focus of interest and no background covariates were adjusted for. Here, we drew on a nationally representative sample of people with MCI and explicitly examined how volunteering was associated with their subsequent cognitive changes.

We addressed this research question by assessing volunteer status (i.e., whether people with MCI volunteered or not) and volunteering dynamics (i.e., how people with MCI transitioned into and out of volunteer roles). Building on extant literature that revealed cognitive benefits of volunteering (Anderson et al., 2014; Guiney & Machado, 2018; Sharifi et al., 2024), we expected volunteering with MCI to be associated with more positive cognitive changes (e.g., flatter cognitive declines or improved cognitive functioning) since baseline. Volunteering dynamics have received less attention in research, but it may be particularly relevant when we study long-term consequences of volunteering. Prior research on volunteering dynamics typically considered four patterns: (a) did not volunteer, (b) initiated volunteering, (c) stopped volunteering, and (d) continuously volunteered (Carr et al., 2018b; Huo & Kim, 2022). A longitudinal study in Sweden found that older adults who continuously volunteered had fewer cognitive complaints than those who never volunteered or discontinued volunteering over 4 years (Griep et al., 2017). Similarly, using data from the HRS from 1998 to 2012, Infurna et al. (2016) tracked 13,262 older Americans who had no cognitive impairment at the initial wave over 14 years. They found that volunteering at baseline and maintenance of volunteering over time were both associated with a reduced likelihood of experiencing cognitive impairment. Group-wise comparisons further suggest that older adults who initiated volunteering after baseline were less likely to develop cognitive impairment than those who never volunteered. Here, we asked whether similar effects were evident in people who already had MCI. We expected having any volunteering experience to be associated with more positive changes in their cognition over time and those who continuously volunteered to exhibit the best cognitive outcomes.

## Other factors

We considered additional sociodemographic characteristics associated with volunteering and cognition, including age, gender, racial/ethnic minority status, marital status, and employment status. The percentage of people volunteering peaks in midlife and then slightly declines with age, which was reported in both the United States and the Netherlands (Broese van Groenou & van Tilburg, 2012; Han et al., 2023). Age also is positively associated with progression from MCI to dementia (Zuliani et al., 2021), which may suggest more severe symptoms with age that could potentially interfere with volunteering. Women typically volunteer more than men do (Krause & Rainville, 2018; Musick & Wilson, 2007).

People from racial/ethnic minority groups tend to volunteer less than their non-Hispanic White counterparts (Johnson & Lee, 2017; U.S. Bureau of Labor Statistics, 2015), which may partly be due to the lack of resources and opportunities available to minority people (Mesch et al., 2006). The racial/ethnic differences can remain the same or be exacerbated in the context of MCI, given racial/ethnic disparities in comorbid conditions and healthcare access (Gupta, 2021; Habibzadeh & Albrecht, 2024). Married or partnered people are more likely to volunteer, since if one spouse volunteers, the other may do too (Mesch et al., 2006). Having a spouse or partner is often accompanied by having a strong social network, which also can expose people to more volunteer opportunities (Dury et al., 2015; Pilkington et al., 2012). It is unclear how employment status may be associated with volunteering with MCI. People who continue to work in the presence of MCI may have more reserved capabilities that would also allow them to volunteer more than those who no longer work, and involvement in multiple roles (an employee and a volunteer) typically contributes to productive aging (Morrow-Howell, 2000). Yet, role strains and conflicts are likely to emerge in the context of MCI.

## The current study

Building on the theoretical framework and prior research, we proposed and tested the following hypotheses among midlife and older adults living with MCI:

*Hypothesis 1:* We expected midlife and older adults with higher SES, a history of volunteer experience, and better physical as well as mental health to be more likely to volunteer in the presence of MCI.

*Hypothesis 2:* We expected midlife and older adults who had any volunteer experience (Hypothesis 2a), especially those who continuously volunteered (Hypothesis 2b), to show more favorable cognitive changes over time.

## Method

### Data and study sample

Data were from the HRS, which examined a nationally representative sample of Americans age 50+ and their spouses (spouses were recruited regardless of age). HRS has been fielded biennially since 1992, but we used data collected between 2002 and 2018. Volunteering activities have been measured since 1998, but some health indicators including functional limitations and depressive symptoms were not assessed until 2002.

Because we were interested in volunteering in the context of MCI and subsequent cognitive outcomes, we identified participants with MCI and tracked them across three waves (T_0_, T_1_, and T_2_; 2 years apart, respectively). We used a researcher contributed dataset (https://hrsdata.isr.umich.edu/data-products/langa-weir-classification-cognitive-function-1995-2020), which includes a total score for participant cognition and derived categories of cognitive impairment based on the Langa–Weir Classifications (Crimmins et al., 2011). Participants completed the Telephone Interview for Cognitive Status (TICS) in each wave and received scores of up to 27 points. A score of 7–11 indicated Cognitive Impairment but no Dementia (CIND), which we referred to as MCI in this study to avoid confusion. Some participants did not complete the TICS themselves, and classification was based on proxy reports. Here, we only selected people who self-completed the TICS, and we defined the wave in which they first met criteria for MCI as T_0_.

A total of 8,433 participants had MCI in any wave between 2002 and 2014 (T_0_), and we excluded 313 participants who were younger than 50 at T_0_. For example, a participant who met criteria for MCI in 2002 (T_0_) was tracked in 2004 (T_1_) and 2006 (T_2_). Likewise, a participant who met criteria for MCI in 2014 (T_0_) was tracked in 2016 (T_1_) and 2018 (T_2_). As such, we considered 2014 as the last possible year for T_0_.

In the remaining sample of 8,120 participants, 6,930 (85%) indicated volunteer status at T_1_ (i.e., whether they volunteered or not within 1 year prior to T_1_), which constituted the final analytic sample for Hypothesis 1. We used participant reports at T_1_ to obtain a proxy of whether they volunteered or not after meeting criterion for MCI at T_0_. Compared to those who did not indicate volunteer status at T_1_ (*n *= 1,190), participants in the final sample were younger, more likely to be female, minority, employed, married, and had better physical, mental, and cognitive health. Notably, among the 6,930 participants who had MCI at T_0_ and indicated volunteer status at T_1_, 6,585 self-completed the TICS at T_1_, whereas 345 were assessed by proxy for their cognition. We compared their TICS scores at T_0_ and found that the 6,585 participants (*M *= 9.77) had significantly higher TICS scores than the 345 participants (*M *= 9.26; *t *= 6.72, *p* < .001). We used data from these 6,585 participants to test Hypothesis 2a.

We additionally considered reports at T_2_ to assess volunteering dynamics (Hypothesis 2b). At T_2_, 5,847 out of the original 6,930 participants were interviewed (469 were alive but did not participate and 614 were deceased). In total, 5,841 participants who provided volunteering reports at both T_1_ and T_2_; these participants were younger, more likely to be female, minority, employed, married, and had better physical, mental, as well as cognitive health, compared to those who provided volunteer status reports at T_1_ but not at T_2_. Further, 5,432 self-completed the TICS at T_2_; again, these 5,432 participants (*M *= 9.79) had higher TICS scores at T_0_ than the 415 participants (*M *= 9.42) who did not complete the TICS at T_2_ (*t *= 5.37, *p* < .001). That is, 5,427 participants provided data on volunteering dynamics and also cognition. We used data from these 5,427 participants to test Hypothesis 2b.

### Measures

#### Volunteering

In each wave, participants indicated whether they had spent any time in the past 12 months doing volunteer work for religious, educational, health-related, or other charitable organizations (1 = *yes*, 0 = *no*). We generated four patterns of volunteering dynamics: (a) not volunteering at either T_1_ or T_2_ (i.e., did not volunteer), (b) not volunteering at T_1_ but volunteering at T_2_ (i.e., initiated volunteering), (c) volunteering at T_1_ but not volunteering at T_2_ (i.e., stopped volunteering), and (d) volunteering at both T_1_ and T_2_ (i.e., continuously volunteered). We assessed volunteer status at T_0_ to measure volunteering history (prior to meeting criterion for MCI in HRS).

#### SES and health

Socioeconomic status indicators included education as the number of years they attended school, household income, and wealth. We used the income and wealth data cleaned and imputed by the RAND Corporation. Due to distribution skewness, income was log-transformed and wealth was transformed using the inverse hyperbolic sine function (Friedline et al., 2015).

For physical health, we considered self-rated health on a scale from 1 (*poor*) to 5 (*excellent*), functional limitations in performing each of the 12 activities (e.g., walking, jogging, and picking up a dime; 1 = *yes*, 0 = *no*), and number of chronic conditions (i.e., hypertension, diabetes, cancer, lung disease, heart disease, stroke, psychiatric problems, and arthritis).

Depressive symptoms were self-reported using the shortened version of the Center for Epidemiologic Studies Depression Scale. There were nine items in the measure before 2014, but one item (“had a lot of energy”) has been omitted ever since. We summed up the number of symptoms each participant had experienced during the past week (1 = *yes*, 0 = *no*) considering eight items that were consistently measured between 2002 and 2014: (a) felt depressed, (b) felt everything was an effort, (c) had restless sleep, (d) was happy, (e) felt lonely, (f) enjoyed life, (g) felt sad, and (h) could not get going. We reverse-coded the two positively phrased items and created a sum for each participant in each wave.

#### Cognition

In each wave, participants completed the TICS (Ofstedal et al., 2005). The interview tested immediate and delayed recall of 10 words (1 point per word), five trials of serial 7 s (1 point per trial), and backward counting (2 points). The composite score of cognition ranges from 0 to 27; higher scores indicate better cognitive functioning. We examined each participant’s cognition scores at T_0_, T_1_, and T_2_.

#### Background characteristics

Participants reported demographic characteristics, including age (in years), gender (1 = *male*, 0 = *female*), racial/ethnic minority status (1 = *Hispanic/Black/other race*, 0 = *non-Hispanic White*), marital status (1 = *married/partnered*, 0 = *nonmarried/partnered*), and employment status (1 = *working for pay*, 0 = *not working for pay*).

### Analytic strategy

Before hypothesis testing, we performed *t* and *χ*^2^ tests to compare volunteers and nonvolunteers in participants with MCI (i.e., depending on whether participants reported volunteering at T_1_ or not). We also ran bivariate correlations linking background characteristics and volunteer status to cognition.

We estimated two-level models using SAS 9.4 to account for the data structure of participants nested within households: 1,482 out of the full sample of 6,930 participants were from the same families (household *n *= 741). We first identified characteristics of people who reported volunteering at T_1_ in the presence of MCI. Because volunteer status was a binary variable, we ran a two-level logistic regression using PROC GLIMMIX to predict the likelihood of volunteering, reporting odds ratios. In this model, we examined resources at T_0_ (i.e., SES, volunteering history, and physical/mental health) as predictors, adjusting for demographic characteristics at T_0_ as covariates.

We then asked how volunteering was associated with subsequent cognitive functioning, while T_0_ cognition was adjusted for (which allowed us to capture residualized changes in cognition; Rovine & Liu, 2012). Because cognition scores were continuous, we ran two-level linear regressions using PROC MIXED. We examined how volunteering between T_0_ and T_1_ (reported at T_1_) was associated with participants’ cognition at T_1_, adjusting for all background characteristics at T_1_ and cognitive scores at T_0_. We also examined how four patterns of volunteering dynamics were associated with participants’ cognition at T_2_, adjusting for all background characteristics at T_2_ and cognitive scores at T_0_. We dummy-coded the volunteering dynamics variable and switched between reference categories for pairwise comparisons.

## Results

Table 1 describes baseline characteristics of participants who met criterion for MCI at T_0_ and comparisons by participants’ volunteer status reported at T_1_. Among the 6,930 participants who had MCI identified at T_0_ and indicated volunteer status at T_1_, 1,690 (24%) reported volunteering at T_1_. Participants who reported volunteering at T_1_ were younger, more likely to be employed and married, and had greater SES and health. These participants were also more likely to report volunteering at T_0_ and T_2_, than participants who reported not volunteering at T_1_. In total, 5,841 participants with MCI provided data on volunteering dynamics: 3,913 reported not volunteering at either T_1_ or T_2_ (i.e., did not volunteer; 67%), 418 reported not volunteering at T_1_ but volunteering at T_2_ (i.e., initiated volunteering; 7%), 561 reported volunteering at T_1_ but not volunteering at T_2_ (i.e., stopped volunteering; 10%), and 949 reported continuously volunteering at T_1_ and T_2_ (i.e., continuously volunteered; 16%). Given the attrition across waves, we present in Supplementary Table 1 (see online supplementary material) the characteristics of subsamples who provided data used for Hypothesis 2a (i.e., those who self-completed the TICS at T_0_ and T_1_; *n *= 6,585) and Hypothesis 2b (those who provided data on volunteering dynamics and self-completed the TICS T_0_ through T_2_; *n *= 5,427), respectively.

**Table 1.** Sample characteristics at baseline (T_0_) and comparisons between participants reporting volunteering and not volunteering at T_1_.

	Full sample		Participated in volunteering		Not participated in volunteering		
	(*N *= 6,930)		(*n *= 1,690)		(*n *= 5,240)		
Variables	*M*	(*SD*)	*M*	(*SD*)	*M*	(*SD*)	*t* or *χ* ^2^
**Background characteristics**							
** Age**	67.93	(11.18)	67.45	(10.55)	68.09	(11.37)	−2.12*
** Male, %**	43		43		43		0.13
** Racial/ethnic minority, %**	44		45		44		0.26
** Married, %**	59		64		57		20.30***
** Employed, %**	29		36		27		49.46***
**Resources**							
** Education**	11.90	(6.55)	12.98	(6.99)	11.56	(6.36)	7.78***
** Income**	49,978.22	(723,636.02)	52,279.28	(90,708.94)	49,236.08	(830,610.16)	0.15
** Wealth**	249,391.88	(696,310.18)	294,050.48	(567,973.96)	234,988.63	(732,392.25)	3.03***
** Self-rated health**	2.86	(1.10)	3.15	(1.06)	2.76	(1.09)	12.99***
** Functional limitations**	3.07	(2.92)	2.33	(2.59)	3.31	(2.98)	−12.98***
** Chronic conditions**	2.12	(1.48)	1.90	(1.40)	2.18	(1.50)	−7.19***
** Depressive symptoms**	2.02	(2.23)	1.54	(1.93)	2.17	(2.30)	−11.06***
**TICS scores**							
** Cognition**	9.74	(1.28)	9.96	(1.20)	9.67	(1.30)	8.55***
**Volunteer status**							
** Volunteered, %**	27		68		13		1,984.59***

*Note*. Participant *N *= 6,930. TICS = Telephone Interview for Cognitive Status; T1 = 2 years after baseline.

* *p* < .05.

*** *p* < .001.

Interestingly, regardless of which subsample we examined, cognitive scores increased from T_0_ to T_1_ (*p* < .001). Yet, participants’ cognitive scores (those who provided cognitive scores at both waves) then decreased from T_1_ to T_2_ (*t *= 10.29, *p* < .001; comparisons not shown in table). Table 2 presents bivariate correlations between sociodemographic characteristics, volunteering, and cognition across waves.

**Table 2.** Bivariate correlations of TICS cognitive scores with background characteristics and volunteer status.

Variables	Cognition T_0_	Cognition T_1_	Cognition T_2_
**Background characteristics**			
** Age T_0_**	.01	−.15***	−.20***
** Male**	.01	−.01	.00
** Racial/ethnic minority**	−.10***	−.09***	−.07***
** Married T_0_**	.05***	.07***	.07***
** Married T_1_**	.06***	.07***	.07***
** Married T_2_**	.05***	.08***	.09***
** Employed T_0_**	.07***	.16***	.20***
** Employed T_1_**	.07***	.15***	.19***
** Employed T_2_**	.07***	.15***	.20***
**Resources**			
** Education**	.07***	.12***	.14***
** Income T_0_**	.02	.02	.01
** Income T_1_**	.04**	.15***	.12***
** Income T_2_**	.06***	.13***	.13***
** Wealth T_0_**	.04**	.07***	.05***
** Wealth T_1_**	.02	.07***	.04**
** Wealth T_2_**	.04**	.09***	.05***
** Self-rated health T_0_**	.08***	.14***	.14***
** Self-rated health T_1_**	.10***	.16***	.16***
** Self-rated health T_2_**	.09***	.14***	.15***
** Functional limitations T_0_**	−.06***	−.12***	−.13***
** Functional limitations T_1_**	−.08***	−.14***	−.14***
** Functional limitations T_2_**	−.08***	−.15***	−.17***
** Chronic conditions T_0_**	−.04**	−.08***	−.10***
** Chronic conditions T_1_**	−.05***	−.08***	−.10***
** Chronic conditions T_2_**	−.06***	−.09***	−.11***
** Depressive symptoms T_0_**	−.07***	−.10***	−.12***
** Depressive symptoms T_1_**	−.09***	−.12***	−.14***
** Depressive symptoms T_2_**	−.09***	−.12***	−.13***
**Volunteer status**			
** Volunteered T_0_**	.08***	.10***	.07***
** Volunteered T_1_**	.10***	.12***	.11***
** Volunteered T_2_**	.09***	.14***	.13***

*Note*. Participant *N *= 6,930. Specific sample sizes vary for different correlations due to attrition. TICS = Telephone Interview for Cognitive Status; T1 = 2 years after baseline; T2 = 4 years after baseline.

** *p* < .01.

*** *p* < .001.

### Resources associated with volunteering in MCI

We estimated a two-level logistic regression to examine how participants’ SES, volunteering history, and physical as well as mental health were associated with the likelihood of volunteering with MCI (see Table 3). Among people meeting criteria for MCI at T_0_, those who attained more years of education (*p* < .001), had greater wealth (*p* = .028), reported better self-rated health (*p* = .003), had fewer functional limitations (*p* = .001), and reported volunteering at T_0_ (*p* < .001) were more likely to report volunteering at T_1_.

**Table 3.** Two-level logistic regression predicting the likelihood of volunteering among people with MCI.

Variables	*B*	(*SE*)	OR
**Fixed effects**			
** Intercept**	−2.18***	(0.39)	—
***Resources***			
** Education**	0.02***	(0.00)	1.02
** Income T_0_**	0.03	(0.02)	1.03
** Wealth T_0_**	0.01*	(0.01)	1.01
** Volunteered T_0_**	2.56***	(0.07)	12.94
** Self-rated health T_0_**	0.11**	(0.04)	1.12
** Functional limitations T_0_**	−0.05***	(0.02)	0.95
** Chronic conditions T_0_**	0.00	(0.03)	1.00
** Depressive symptoms T_0_**	−0.03	(0.02)	0.97
***Background characteristics***			
** Age T_0_**	−0.01**	(0.00)	0.99
** Male**	−0.09	(0.07)	0.91
** Racial/ethnic minority**	0.15	(0.08)	1.16
** Married T_0_**	0.07	(0.08)	1.07
** Employed T_0_**	0.04	(0.08)	1.04
**Random effects**			
** Intercept VAR (Level 2)**	0.05	(0.11)	—
**−2 (pseudo) log likelihood**	34,269.1		

*Note*. Participant *N *= 6,930. MCI = mild cognitive impairment; OR = odds ratio; T0 = baseline; VAR = variance.

* *p* < .05.

** *p* < .01.

*** *p* < .001.

### Volunteering and subsequent cognitive changes in MCI

We then estimated two-level regression models to examine how volunteer status and dynamics were associated with subsequent cognitive changes among participants who met criterion for MCI at T_0_ (see Table 4). Volunteering reported at T_1_ (i.e., engaging in volunteering within 1 year prior to T_1_) was associated with a greater increase in cognitive scores between T_0_ and T_1_ (*p* < .001). As for volunteering dynamics (see Table 5), we found that participants who continuously volunteered (*p* < .001) or initiated volunteering (*p* = .012) reported greater increases in cognition than those who did not volunteer. Participants who continuously volunteered did not differ from those who initiated volunteering (*p* < .18), but they reported greater increases in cognition than those who stopped volunteering (*p* = .003). There was no difference between participants who stopped volunteering and those who did not volunteer (*p* = .31), or between participants who initiated volunteering and those who stopped (*p* = .21).

**Table 4.** Two-level regression model predicting residualized changes in cognitive scores between T_0_ and T_1_ from volunteer status at T_1_.

Variables	*B*	(*SE*)
**Fixed effects**		
** Intercept**	8.69***	(0.60)
** Volunteered T_1_**	0.54***	(0.11)
***Covariates***		
** Age T_1_**	−0.08***	(0.01)
** Male**	−0.35***	(0.09)
** Racial/ethnic minority**	−0.93***	(0.10)
** Married T_1_**	−0.18	(0.10)
** Employed T_1_**	0.35**	(0.12)
** Education**	0.04***	(0.01)
** Income T_1_**	0.19***	(0.03)
** Wealth T_1_**	0.03***	(0.01)
** Self-rated health T_1_**	0.26***	(0.05)
** Functional limitations T_1_**	−0.02	(0.02)
** Chronic conditions T_1_**	0.06	(0.03)
** Depressive symptoms T_1_**	−0.10***	(0.02)
** Cognition T_0_**	0.65***	(0.04)
**Random effects**		
** Intercept VAR (Level 2)**	0.72	(0.50)
** Residual VAR**	12.39***	(0.54)
**−2 log likelihood**	35,500.7	

*Note*. Participant *n *= 6,585. T0 = baseline; T1 = 2 years after baseline; VAR = variance.

* *p* < .05.

** *p* < .01.

*** *p* < .001.

**Table 5.** Two-level regression models predicting residualized changes in cognitive scores from patterns of volunteering dynamics between T_0_ and T_2_.

	Model 1		Model 2		Model 3		Model 4	
Variables	*B*	(*SE*)	*B*	(*SE*)	*B*	(*SE*)	*B*	(*SE*)
**Fixed effects**								
** Intercept**	11.35***	(0.68)	11.84***	(0.70)	11.53***	(0.70)	12.14***	(0.70)
** Did not volunteer**	[Ref.]		−0.49*	(0.19)	−0.18	(0.18)	−0.79***	(0.14)
** Initiated volunteering**	0.49*	(0.19)	[Ref.]		0.31	(0.25)	−0.30	(0.22)
** Stopped volunteering**	0.18	(0.18)	−0.31	(0.25)	[Ref.]		−0.61**	(0.20)
** Continuously volunteered**	0.79***	(0.14)	0.30	(0.22)	0.61**	(0.20)	[Ref.]	
***Covariates***								
** Age T_2_**	−0.10***	(0.01)	−0.10***	(0.01)	−0.10***	(0.01)	−0.10***	(0.01)
** Male**	−0.36***	(0.11)	−0.36***	(0.11)	−0.36***	(0.11)	−0.36***	(0.11)
** Racial/ethnic minority**	−0.91***	(0.12)	−0.91***	(0.12)	−0.91***	(0.12)	−0.91***	(0.12)
** Married T_2_**	−0.07	(0.11)	−0.07	(0.11)	−0.07	(0.11)	−0.07	(0.11)
** Employed T_2_**	0.61***	(0.14)	0.61***	(0.14)	0.61***	(0.14)	0.61***	(0.14)
** Education**	0.05***	(0.01)	0.05***	(0.01)	0.05***	(0.01)	0.05***	(0.01)
** Income T_2_**	0.15***	(0.03)	0.15***	(0.03)	0.15***	(0.03)	0.15***	(0.03)
** Wealth T_2_**	0.04***	(0.01)	0.04***	(0.01)	0.04***	(0.01)	0.04***	(0.01)
** Self-rated health T_2_**	0.13*	(0.06)	0.13*	(0.06)	0.13*	(0.06)	0.13*	(0.06)
** Functional limitations T_2_**	−0.06**	(0.02)	−0.06**	(0.02)	−0.06**	(0.02)	−0.06**	(0.02)
** Chronic conditions T_2_**	0.01	(0.04)	0.01	(0.04)	0.01	(0.04)	0.01	(0.04)
** Depressive symptoms T_2_**	−0.12***	(0.03)	−0.12***	(0.03)	−0.12***	(0.03)	−0.12***	(0.03)
** Cognition T_0_**	0.52***	(0.04)	0.52***	(0.04)	0.52***	(0.04)	0.52***	(0.04)
**Random effects**								
** Intercept VAR (Level 2)**	1.07*	(0.60)	1.07*	(0.60)	1.07*	(0.60)	1.07*	(0.60)
** Residual VAR**	12.48***	(0.64)	12.48***	(0.64)	12.48***	(0.64)	12.48***	(0.64)
**−2 log likelihood**	29,448.8		29,448.8		29,448.8		29,448.8	

*Note*. Participant *n *= 5,427. T0 = baseline; T2 = 4 years after baseline; VAR = variance.

* *p* < .05.

** *p* < .01.

*** *p* < .001.

## Discussion

Research has documented positive effects of volunteering in later years (Anderson et al., 2014; Guiney & Machado, 2018; Sharifi et al., 2024), but we uniquely contributed to the literature by revealing its cognitive benefits in midlife and older adults living with MCI—a vulnerable population at elevated risk for ADRD. About one in four people with MCI volunteered, and our results linked greater socioeconomic resources, a history of volunteering, and better physical health to a greater likelihood of volunteering with MCI. Yet, regardless of sociodemographic characteristics, those who volunteered, particularly those who continuously volunteered or initiated volunteering in MCI, exhibited more positive cognitive changes. Overall, our results offer support to the conceptual framework we proposed, identifying how resources drive individual variation in volunteering with MCI and delineating the lasting cognitive benefits of volunteering in this unique context. This study joins the mounting work on volunteering and cognitive health (Guiney & Machado, 2018; Sharifi et al., 2024) and offers invaluable empirical evidence that informs tailored volunteer programs targeting those who need cognitive gains the most (Klinedinst & Resnick, 2016; Lissek & Suchan, 2021; Piette et al., 2023a, 2023b).

### Resources associated with volunteering in MCI

Based on bivariate correlations and fully adjusted models, SES, volunteering history, and physical health consistently predicted volunteering in the context of MCI, which aligns with and extends theories and research primarily focused on the general population (Dury et al., 2015; Musick & Wilson, 2007). Indeed, people with higher SES backgrounds may have easier access to volunteer opportunities matched to their compromised capabilities. They also receive more timely treatments than their lower SES counterparts (Qian et al., 2014) that can better reserve the necessary capabilities to volunteer.

Adding to previous research (Tang et al., 2010), we found that people with prior volunteer experience were more likely to be sustain their volunteer roles, even when they had to be adjusting to MCI. These people typically feel better supported, more committed, and more informed of various volunteer opportunities. They may also have a better understanding of what volunteer activities involve, which allows them to continue making contributions in ways they can. In this study, we were only able to measure volunteer activities right before participants met criteria for MCI and not able to know whether they volunteered earlier (e.g., in young adulthood). Our result suggests continuity in volunteer activities when MCI occurs, but future research may explore how prior experience affects the way people with MCI views volunteering.

People with better physical health (based on self-rated physical health and functional limitations) may be more able to volunteer and engage in activities that are not specifically tailored for those with MCI, thereby accessing more opportunities. Interventions designed for people with MCI tend to be sedentary and less physically demanding (Klinedinst & Resnick, 2016; Piette et al., 2023a, 2023b). The number of chronic conditions does not necessarily limit people’s functional capabilities (Verbrugge & Jette, 1994) and thus may not be associated with volunteering.

Volunteers and nonvolunteers likely differ in other common correlates of volunteering, such as depressive symptoms, marital status, and employment status as shown in bivariate correlations. Yet, they are not robust predictors of volunteering in the context of MCI. For example, even if a married person with MCI shows a stronger desire to volunteer than a single person with MCI, actual engagement in volunteer activities may still depend on their resources and physical health.

### Volunteering and subsequent cognitive changes in MCI

We found robust associations between volunteering and more positive cognitive changes among midlife and older adults with MCI. This finding adds to extant literature on volunteering that has primarily focused on cognitively healthy older adults and offers empirical support to theories linking volunteering to reduced dementia risk (Anderson et al., 2014; Guiney & Machado, 2018; Sharifi et al., 2024). Involving people with MCI in volunteer activities holds promise for slowing their cognitive declines and protecting this vulnerable population.

Moreover, we assessed volunteering dynamics, as older adults, especially those living with MCI, likely transition out of volunteer roles at some point. Our observation was in line with prior work tracking older adults who had no cognitive impairment at baseline (Griep et al., 2017; Infurna et al., 2016), showing that those who continuously volunteered exhibited better cognitive outcomes than those who never volunteered or stopped volunteering. We uniquely identified benefits of the initiation of volunteering in the presence of MCI, in that people who began volunteering a few years after experiencing MCI had similar cognitive changes as those who continuously volunteered. A burgeoning literature has revealed how initiating volunteering leads to better changes in physical and mental health, even in the face of stressful life transitions such as widowhood (Carr et al., 2018a, 2018b). We add to this work with a focus on older adults’ cognitive health.

Overall, our findings highlight the importance of sustaining people with MCI who already volunteer and encouraging those who have not yet—both of which call for more tailored volunteer opportunities that are accessible in the context of MCI. Indeed, a study with older residents in continuing care retirement communities has found that sometimes interests in volunteering are dampened by challenges such as a lack of access to appropriate volunteer opportunities (Resnick et al., 2013).

### Limitations and conclusion

We acknowledge some limitations. MCI was detected based on a single cognitive test. TICS is a validated tool for remotely detecting MCI (Chappelle et al., 2023), but participants’ cognitive scores could have been affected by other factors such as health status on the interview day. Some participants may have transitioned in and out of MCI status throughout their study participation, but we treated their first time meeting criteria for MCI as the baseline. As we acknowledged earlier, attrition occurred; some participants were deceased or unable to self-complete the TICS and they had lower cognitive scores at baseline than those who stayed in the study. Findings should be interpreted with caution, as the data do not allow for causal claims. Participants in the HRS reported their volunteering activities within 1 year prior to their biennial core interviews. As such, we might have underestimated engagement in volunteering among older adults with MCI. If a participant meeting criteria for MCI at T_0_ volunteered within 1 year after T_0_ but not in the following year (i.e., 1 year prior to T_1_—a period that T_1_ volunteering report corresponded to), this participant was considered as not volunteering. We also did not have data to test the specific types of volunteer activities that our participants engaged in, which mattered substantially in the context of MCI and comorbid conditions. Future studies may gather more detailed information to better guide the development of interventions targeting people with MCI. Further, although it remains unclear why volunteering improves cognitive health in people with MCI, this study creates a foundation for future work in this exciting field. One interesting future direction relies on qualitative research that may better capture how these people perceive and engage in volunteering as they continue to explore meaning in life and make contributions to their communities.

This study presents a novel investigation that explicitly identified midlife and older adults who volunteered in the presence of MCI and tracked their subsequent cognitive changes over time. Findings advance our understanding of volunteering and cognitive health in late life when pathological cognitive impairment occurs (Lee et al., 2021). More importantly, we join the growing body of work that designs volunteer programs targeting people with MCI. Although existing programs are small-scale and selective, they have confirmed the feasibility of having people with MCI use their reserved capabilities to help others in small but meaningful ways (e.g., make quilts for sick children, read to people who have poor vision, visit isolated residents at assisted living). Our work identifies people who may need additional resources to access volunteer opportunities in the presence of MCI and corroborates the cognitive benefits that people with MCI can obtain from these volunteer activities. It can be financially demanding to design and sustain the implementation of volunteer interventions for people with MCI (Svedin et al., 2023). For example, given the various, unique limitations faced by people with MCI, these intervention programs may require dedicated staff to supervise volunteers, respond to volunteers’ emergent cognitive or health changes, and coordinate across healthcare and community settings. Yet, our study points out the potential of volunteering as a feasible strategy to slow cognitive declines in midlife and older adults with MCI and protect them against ADRD. Continued work in this area is warranted to better identify the mechanisms underlying the benefits of volunteering in this context and strengthen the evidence base that may attract more consistent funding streams.

## Supplementary Material

gnaf190_Supplementary_Data

## Data Availability

The current study drew on data from the *Health and Retirement Study* (HRS), which are publicly available on the HRS website (https://hrsdata.isr.umich.edu/data-products/public-survey-data?_ga=2.124584909.696739851.1613501184-1622400632.1584043405). The data we used for analysis and our analytic methods are described in detail in the text and will be made available to other researchers for replication purposes upon request. This study did not involve clinical trials and was not preregistered.
