# Supplementary material for: Volunteering with mild cognitive impairment: implications for subsequent cognitive changes
Source: Gerontologist. 2025 Sep 10;65(11):gnaf190. doi: 10.1093/geront/gnaf190 (PMC12596150; doi:10.1093/geront/gnaf190)
Supplement: gnaf190_Supplementary_Data [file gnaf190_supplementary_data.zip › Huo & Kim Suppl.docx]

**Supplementary Material**

**Supplementary Table 1**

*Sample Characteristics for Participants who Provided Data for Hypotheses 2a and 2b*

|  | Sample for Hypothesis 2a  (*n* = 6,585) | |  | Sample for Hypothesis 2b  (*n* = 5,427) | |
| --- | --- | --- | --- | --- | --- |
| Variables | *M* | (*SD*) |  | *M* | (*SD*) |
| **Background characteristics** |  |  |  |  |  |
| Age T_0_ | 67.59 | (11.05) |  | 66.55 | (10.60) |
| Male, % | 43 |  |  | 42 |  |
| Racial/ethnic minority, % | 45 |  |  | 47 |  |
| Married T_0_, % | 59 |  |  | 59 |  |
| Married T_1_, % | 56 |  |  | 57 |  |
| Married T_2_, % | 54 |  |  | 55 |  |
| Employed T_0_, % | 30 |  |  | 32 |  |
| Employed T_1_, % | 26 |  |  | 29 |  |
| Employed T_2_, % | 23 |  |  | 24 |  |
| **Resources** |  |  |  |  |  |
| Education | 11.90 | (6.49) |  | 11.85 | (6.59) |
| Income T_0_ | 50,046.63 | (742,062.80) |  | 52,084.20 | (816,314.15) |
| Income T_1_ | 40,911.00 | (72,753.94) |  | 41,395.98 | (76,458.56) |
| Income T_2_ | — | — |  | 41,289.49 | (71,962.08) |
| Wealth T_0_ | 243,565.00 | (688,671.21) |  | 236,666.13 | (714,779.00) |
| Wealth T_1_ | 258,533.49 | (841,040.07) |  | 255,589.17 | (877,351.79) |
| Wealth T_2_ | — | — |  | 264,596.35 | (980,750.57) |
| Self-rated health T_0_ | 2.86 | (1.10) |  | 2.89 | (1.10) |
| Self-rated health T_1_ | 2.82 | (1.09) |  | 2.86 | (1.09) |
| Self-rated health T_2_ | — | — |  | 2.81 | (1.09) |
| Functional limitations T_0_ | 3.05 | (2.92) |  | 2.93 | (2.88) |
| Functional limitations T_1_ | 3.28 | (2.98) |  | 3.13 | (2.93) |
| Functional limitations T_2_ | — | — |  | 3.33 | (2.98) |
| Chronic conditions T_0_ | 2.10 | (1.47) |  | 2.01 | (1.44) |
| Chronic conditions T_1_ | 2.34 | (1.53) |  | 2.24 | (1.50) |
| Chronic conditions T_2_ | — | — |  | 2.47 | (1.55) |
| Depressive symptoms T_0_ | 2.02 | (2.23) |  | 2.02 | (2.25) |
| Depressive symptoms T_1_ | 1.98 | (2.24) |  | 1.95 | (2.24) |
| Depressive symptoms T_2_ | — | — |  | 1.93 | (2.21) |
| **TICS scores** |  |  |  |  |  |
| Cognition T_0_ | 9.77 | (1.27) |  | 9.96 | (1.20) |
| Cognition T_1_ | 12.16 | (3.94) |  | 12.99 | (3.86) |
| Cognition T_2_ | — | — |  | 12.62 | (3.94) |
| **Volunteer status** |  |  |  |  |  |
| Volunteered T_0_, % | 27 |  |  | 28 |  |
| Volunteered T_1_, % | 25 |  |  | 27 |  |
| Volunteered T_2_, % | — |  |  | 25 |  |

*Note*. TICS = Telephone Interview for Cognitive Status. The sample for Hypothesis 2a included participants who indicated volunteer status and self-completed the TICS at T_1_. The sample for Hypothesis 2b included participants who provided data on volunteering dynamics (indicating volunteer status at both T_1_ and T_2_) and self-completed the TICS at T_2_.
